# Accurate Prediction of NMR Chemical Shifts: Integrating DFT Calculations with Three-Dimensional Graph Neural Networks

**DOI:** 10.1021/acs.jctc.4c00422

**Published:** 2024-06-06

**Authors:** Chao Han, Dongdong Zhang, Song Xia, Yingkai Zhang

**Affiliations:** † Department of Chemistry, New York University, New York, New York 10003, United States; ‡ Simons Center for Computational Physical Chemistry at New York University, New York, New York 10003, United States; § NYU-ECNU Center for Computational Chemistry at NYU Shanghai, Shanghai 200062, China

## Abstract

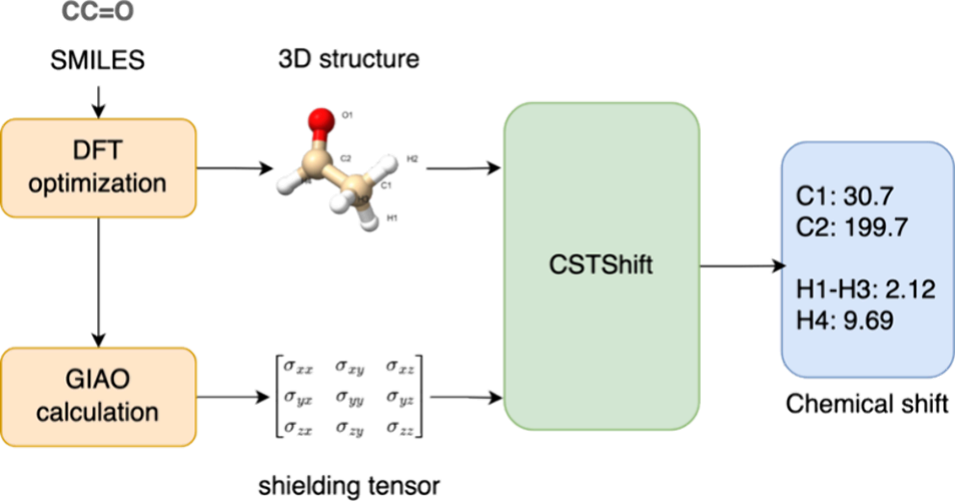

Computer prediction of NMR chemical shifts plays an increasingly important role in molecular structure assignment and elucidation for organic molecule studies. Density functional theory (DFT) and gauge-including atomic orbital (GIAO) have established a framework to predict NMR chemical shifts but often at a significant computational expense with a limited prediction accuracy. Recent advancements in deep learning methods, especially graph neural networks (GNNs), have shown promise in improving the accuracy of predicting experimental chemical shifts, either by using 2D molecular topological features or 3D conformational representation. This study presents a new 3D GNN model to predict ^1^H and ^13^C chemical shifts, CSTShift, that combines atomic features with DFT-calculated shielding tensor descriptors, capturing both isotropic and anisotropic shielding effects. Utilizing the NMRShiftDB2 data set and conducting DFT optimization and GIAO calculations at the B3LYP/6-31G(d) level, we prepared the NMRShiftDB2-DFT data set of high-quality 3D structures and shielding tensors with corresponding experimentally measured ^1^H and ^13^C chemical shifts. The developed CSTShift models achieve the state-of-the-art prediction performance on both the NMRShiftDB2-DFT test data set and external CHESHIRE data set. Further case studies on identifying correct structures from two groups of constitutional isomers show its capability for structure assignment and elucidation. The source code and data are accessible at https://yzhang.hpc.nyu.edu/IMA.

## Introduction

Nuclear magnetic resonance (NMR) spectroscopy stands as an indispensable tool in various domains of chemistry and biology.^1−7^ NMR chemical shifts, which represent the resonance frequency variation of spin-active nuclei due to distinct atomic local environments, are pivotal for determining molecular connectivity, stereochemistry, and conformation.^8−11^ The computational prediction of chemical shifts plays an increasingly important role in the structure elucidation of small organic molecules, especially when the chemical shift cannot be readily assigned from complex NMR spectra. Although density functional theory (DFT) calculations, enhanced by the gauge-including atomic orbital (GIAO) method, have paved the way for structural assignment and elucidation by predicting NMR chemical shift values,^12−19^ their accuracy is limited despite the significant computational resources required. Empirical scaling has been widely applied to improve NMR chemical shift predictions,^17^ in which a linear regression approach is used. The demonstrated accuracy enhancement underscores the potential of integrating more sophisticated, data-driven techniques in the NMR chemical shift prediction.

With the significant advancement of machine learning (ML) techniques and the accumulation of experimental data on NMR chemical shifts in recent years, ML has demonstrated success in the data-driven prediction of NMR chemical shifts for small molecules, solids, and proteins.^20−31^ Particularly, graph neural networks (GNNs) have showcased their superior ability in representation learning across various chemical and biological tasks without the need for precomputed molecular descriptors or fingerprints, operating in an end-to-end manner.^32,33^ GNNs have found wide applications in chemistry and biology, encompassing molecular property prediction,^34−39^ molecule generation,^40−42^ and the prediction of NMR chemical shift values.^43−55^ Molecules can be modeled as either two-dimensional (2D) graphs, where edges represent chemical bonds connecting the atoms, or as three-dimensional (3D) graphs, where edges of interatomic distances provide more intricate conformational information. Jonas and Kuhn employed a 2D convolutional graph neural network to predict ^1^H and ^13^C chemical shifts along with uncertainties.^48^ Subsequent works improved the design of 2D GNNs, resulting in enhanced performance.^44,45,50^ In comparison with models using 2D topological graphs, 3D GNNs take into account more refined geometric factors, leading to superior performance than 2D GNNs when predicting molecular properties like molecular energies.^34,37^ Recent studies have developed 3D GNNs for predicting NMR chemical shifts of small organic molecules and proteins.^43,46^ However, direct comparisons between new 3D GNNs and 2D models are still required to illustrate the necessity of introducing molecular geometry information into the neural network. One novel study by Gao et al. constructed a 3D GNN by combining atomic embedding with calculated isotropic shielding constants to predict experimental chemical shifts in the few-shot setting, with a very small data set of 476 ^13^C and 217 ^1^H experimental chemical shifts including both training and test sets.^51^ Their DFT calculations employed M062X/6-31+G(d,p) for geometry optimization and mPW1PW91/6-311+G(2d,p) for the NMR GIAO calculation. Such a high level of DFT calculations is often recommended for the empirical scaling approach (DFT-LR) to predict NMR chemical shifts and is suitable for few-shot learning and small data sets but would be time-consuming for large molecules and for labeling a much bigger data set. Thus, it remains to be explored whether the combination of DFT-calculated NMR information and 3D GNN-learned atomic representation would be a promising approach to achieving accurate prediction of chemical shifts with a much larger data set. In addition, to make such an approach more applicable, the computational cost should also be controlled at an affordable level.

In this work, a 3D GNN model incorporating atomic descriptors from B3LYP/6-31G*-optimized geometries and calculated shielding tensors (CSTs) has been developed to predict ^1^H and ^13^C chemical shifts for small molecules to achieve state-of-the-art performance. CST descriptors are three normalized eigenvalues of the DFT-calculated NMR shielding tensors for all atoms within a given molecule, providing atomic environmental information derived from the electronic structure. Rooted in the sPhysNet architecture,^56,57^ our model CSTShift includes DFT-calculated NMR information by concatenating CST descriptors with atomic representations in the GNN. Based on the NMRShiftDB2^58,59^ data set containing experimental NMR chemical shifts for ^1^H and ^13^C of over 40,000 molecules, DFT optimization and GIAO calculations at the level of B3LYP/6-31G(d) were conducted to prepare a large data set NMRShift2DB2-DFT comprising computational information and experimental NMR data for model development. With DFT-optimized 3D structures and the incorporation of calculated shielding tensors, our model reduced prediction errors to mean absolute error (MAE) values of 0.944 ppm on ^13^C and 0.185 ppm on ^1^H. CSTShift also outperformed the traditional DFT/GIAO-LR (linear regression) model and previous 3D GNN model on the CHESHIRE data set, achieving MAE values of 0.504 ppm on ^13^C and 0.078 ppm on ^1^H. Last, to demonstrate our model’s ability in structural elucidation, we directly tested it on two groups of constitutional isomers, which exhibited superior performance compared to other reported models.

### Data Sets

#### NMRShiftDB2-DFT

**Figure 1:**
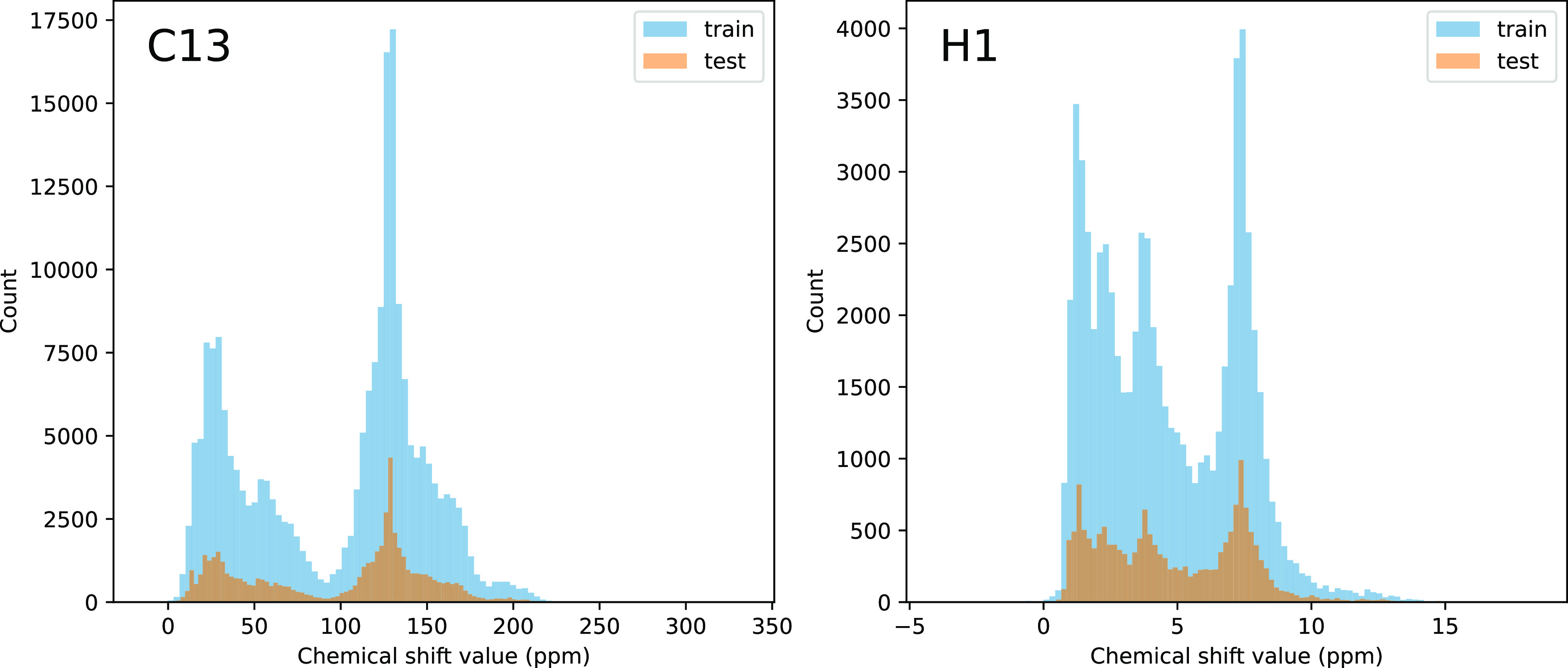
Data distribution of chemical shifts for ^13^C (left) and ^1^H (right) in NMRShiftDB2.

We conducted DFT optimization and NMR calculation to prepare the NMRShiftDB2-DFT data set for model training based on the NMRShiftDB2 data set revision 1624, accessible via https://sourceforge.net/p/nmrshiftdb2/code/1624/. This experimental data set was initially released by Kuhn et al.^58,59^ and further processed by their subsequent work.^48^ The original NMRShiftDB2 contains over 40,000 compounds with ^1^H and ^13^C chemical shifts from NMR spectra measured under different solvent and temperature conditions. The processed version excluded molecules containing rarely occurred elements, those failing to pass the sanitize check in RDKit,^60^ or those with more than 64 atoms. A total of 26,913 molecules with ^13^C chemical shifts and 12,806 molecules with ^1^H chemical shifts were selected, limited to elements of C, H, O, N, S, Cl, P, and F. It is worth noting that some molecules have multiple entries either in the train set or test set due to their chemical shift records of ^1^H and ^13^C being obtained from different measured spectra. We adopted this processed version and conducted geometry optimization and NMR calculations, as reported in a later section. The data distribution for ^13^C and ^1^H in the provided train/test set from NMRShiftDB2 is shown in Figure 1.

To equip molecules with 3D conformations, we applied three steps to generate and optimize structures of compounds in the NMRShiftDB2 data set. Given a molecule with its SMILES or RDKit mol object, a maximum of 300 conformers were generated using ETKDG^61^ and optimized with an MMFF94^62^ force field. After removing similar conformers using Butina clustering^63^ with a 0.2 Å RMSD cutoff, five conformers with the lowest MMFF energies for each molecule were optimized in the solvent phase (chloroform) using B3LYP/6-31G(d)^64^ by Gaussian.^65^ All conformers were optimized if the total amount was less than 5. In addition, charged molecules with a small amount were excluded. As only atom species are used for the initialization of the atomic embeddings, molecules with the same atom sets but different charges cannot be distinguished by the model. Among the DFT-optimized conformers, the one with the lowest energy was considered the final DFT-generated 3D structure for the molecule.

**Figure 2:**
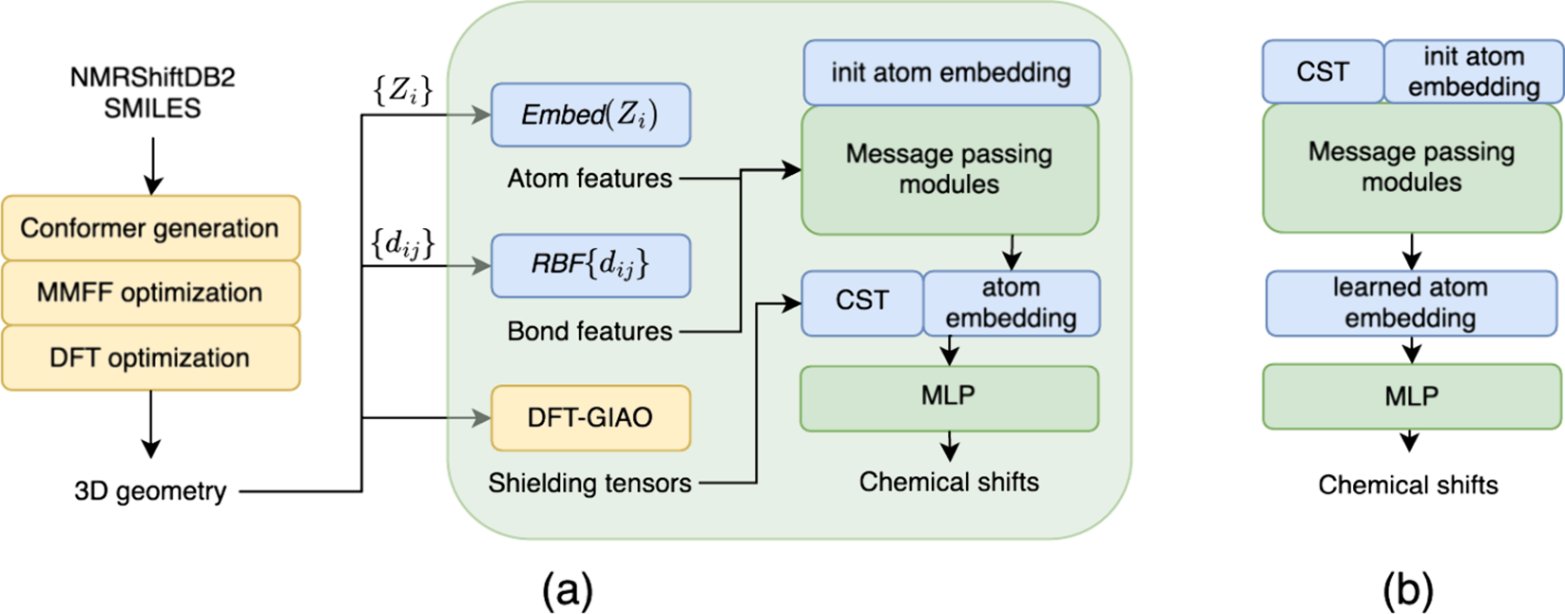
(a) Workflow of CSTShift to predict the NMR chemical shift for organic molecules. Molecular structures are generated by the three-step calculation. As a GNN model, CSTShift takes 3D geometry as input and then embeds atom type {*Z*_*i*_} and interatomic distances {*d*_*ij*_} using atomic embedding layers (Embed) and radial basis function (RBF). The atom embedding is updated in message-passing modules iteratively before being concatenated with CST descriptors derived from DFT-GIAO calculation based on previous optimized 3D structures. The concatenated embedding is fed into fully connected layers (MLP) to provide the final prediction of chemical shifts. (b) Alternative model architecture named CSTShift-emb in contrast to CSTShift-out in panel (a). For CSTShift-emb, CST descriptors are concatenated directly with initialized atom embeddings before message-passing modules. Detailed architecture is shown in Figure S1 in the Supporting Information.

To include additional information from NMR calculation, we construct the atomic CST descriptors using shielding tensors computed by the DFT-GIAO^12^ method. The calculated shielding tensors of a nucleus describe the electronic shielding effect in the external magnetic field.^66^ The average values of these 2-rank shielding tensor eigenvalues are often used to predict the experimental chemical shifts with empirical linear scaling.^10,17^ Strongly correlated to the chemical shifts, shielding tensors could provide great auxiliary information to update atomic embeddings in GNNs. The CST descriptors consist of three eigenvalues of the shielding tensors, including both isotropic and anisotropic effects. The descriptors are normalized for each element individually to provide element-specific information and achieve magnitude alignment with embeddings from previous network layers. Subsequently, these three-dimensional descriptors are concatenated with atomic embeddings, as shown in Figure 2.

To calculate the shielding tensors, the DFT-generated 3D structure undergoes NMR single-point calculation with B3LYP/6-31G(d) and the SMD continuum solvation model.^67^ If solvent information from the experimental measurements for the compounds is explicitly provided, then the solvation modeling is based upon the given solvent. Otherwise, chloroform is used since it is the most used solvent in experimental measurements.

#### CHESHIRE

The CHESHIRE data set was first created by Rablen et al.^68^ and expanded by Tantillo et al.^17^ It served as a benchmark for a series of DFT-based methods with different solvents and solvation models. In the CHESHIRE data set, the test set containing 80 small organic molecules was fitted by linear regression to provide scaling factors, while the probe set containing 25 small organic molecules was used to evaluate the fitting performance. Herein, the CHESHIRE test set serves as an external test set to evaluate the prediction performance.

## Methods

### Model Architecture

We developed the graph neural network named CSTShift based on the PhysNet^57^ and sPhysNet models^56^ to predict chemical shifts, the workflow of which is shown in Figure 2. Taking 3D molecular structures optimized from DFT calculations as input, our model can effectively encode atomic environment information through Gaussian expansion of interatomic distances to update atom embeddings. Additionally, shielding tensors from DFT-GIAO calculations are concatenated with the atom embeddings to provide auxiliary information and improve the final prediction. Different solvent conditions are considered using implicit solvent modeling in the DFT optimization and NMR-GIAO calculations, while other experimental conditions, including temperatures, are not included in CSTShift models.

CSTShift constructs initial atom embeddings based solely on the atom species and initial bond features by expanding atomwise distances via radius basis functions (RBFs) from 3D molecular structures. The atom embeddings  are updated iteratively based on the framework of the message-passing neural network (MPNN):^69^
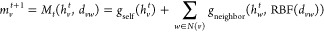

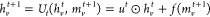
where  is the initial atom features, *g*_self_ is an activation-first linear layer, *g*_neighbor_ is a neural network calculating the interaction from *h*_*w*_ to *h*_*v*_ depending on the RBF expansion of the interatomic distance *d*_*vw*_ between atoms *w* and *v*, *u*^*t*^ is a learnable parameter vector, and *f* is a neural network consisting of one residual layer and one linear layer. The architecture is implemented using PyTorch^70^ and Torch-geometric frameworks.^71^ Detailed hyperparameters can be found in Table S1 in the Supporting Information.

To further introduce auxiliary information for more accurate GNN predictions, we constructed CST (calculated shielding factor) descriptors from DFT NMR calculations. The components of the shielding tensor for a resonant nucleus *N* are calculated as the energy derivatives in the form of , where *E* is the molecular energy, *B* is the external magnetic field, and μ_*N*_ is the nuclear magnetic moment for the atom *N*. The indices *i* and *j* run through 3 directions. Three eigenvalues of these 3 × 3 matrices for each nucleus are used as the CST descriptors of the corresponding atom after element-wise normalization.

By integration of messages from the atomic environment, the atom embeddings are concatenated with the element-wise normalized CST descriptors, in which DFT calculations directly provide additional information for each atom. Subsequently, the atomic readout layer *r* reduces the embedding dimension and generates the final prediction labels for each atom:
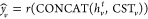


The loss function takes the predicted *y*_*v*_^ with the true chemical shifts on the NMR-active atoms to calculate the mean absolute error (MAE) and update the model’s weights.

To explore the best architecture for leveraging atomic information from DFT calculations, we developed another variant model combining normalized CST descriptors and atom embedding. After initialization, atom embeddings are directly concatenated with CST before the following message-passing steps and output layers. This earlier concatenation enables atomic shielding tensors to revise embeddings for all atoms, while concatenation after message-passing updating keeps more direct influence from CST descriptors and maintains the capability for further transfer learning strategies. We explore the performance of these two implementations and present them in Results and Discussion.

### Training and Evaluation Protocols

Our model is trained on the NMRShiftDB2-DFT data set to learn experimental chemical shifts. For comparison with other models, we divide our screened data set using the training and test split from the previous work^48^ after removing molecules, which failed to obtain DFT-optimized structures. We reserved 5% of molecules from the training data set as the validation set. During training, the model parameters are updated using an AMSGrad^72^ optimizer with an initial learning rate of 0.001. The learning rate is scheduled by ReduceLROnPlateau with the decay factor being 0.5 and the patience epoch being 30. The MAE error on the validation data set is used to adjust the learning rate and stop training when the learning rate reaches 5 × 10^–8^. Typically, fewer than 500 epochs are needed to complete the training.

The CHESHIRE data set serves as an external test data set to evaluate the model’s ability to correctly assign chemical shifts for molecules. Two groups of constitutional isomers, TIC-10^73^ and NHP,^74^ are used to further illustrate our model’s ability for structure elucidation. It is important to note that when measuring our model’s performance using MAE or root-mean-squared error (RMSE), the computation is done on the NMR-active atoms.

## Results and Discussion

### Models Trained on NMRShiftDB2-DFT

During the generation of 3D structures by MMFF and DFT calculation, failed or charged molecules are excluded from the data set. Overlapping molecules in the CHESHIRE data set are also removed. In the reduced data set, the multiplicity still exists, where one molecule structure corresponds to several chemical shift records. While many are caused by measurements in different situations such as in different solvents, we notice that there is a portion of molecules sharing the same geometry and nonconflicting chemical shifts. Reasons might include repeating experiment records or partially examining target atom sets in different trials. We keep one unified record for each set to avoid biased training. The total process removed 2% ^13^C samples and 6% ^1^H samples, as shown in Table 1.

**Table 1:** Molecule Counts in the Original NMRShiftDB2 and Processed NMRShiftDB2-DFT Data Set^a^

**data set**	**data split**	^**13**^**C**	^**1**^**H**
NMRShiftDB2	train	21,523	10,252
	test	5390	2554
NMRShiftDB2-DFT	train	21,064	9590
	test	5289	2393

a Statistics of the original data set comes from the screened version, in which molecules have passed the sanitize check and are restricted by element types and number of atoms.^48^ The processed NMRShiftDB2-DFT data set is used for the training and evaluation.

We first compared our results to other reported results predicting ^13^C and ^1^H chemical shifts on NMRShiftDB2. Baseline models include the HOSE method and three graph neural networks. HOSE used atomic featurization and the nearest-neighbor approach to predict chemical shifts.^75^ All the baseline graph neural networks embedded molecules using 2D features. To demonstrate individual contributions, we designed three variants of the CSTShift model: concatenating CST descriptors after embedding initialization (CSTShift-emb), concatenating before the output layers (CSTShift-out), and no concatenation (CSTShift-noCST). Finally, we proposed ensemble models of two variants with concatenation to further improve the performance. Five models are trained individually with random initialization and identical hyperparameters. The average prediction is used as the final ensemble prediction.

The performance comparison in Table 2 shows that the CSTShift model improves the prediction accuracy for ^13^C and achieves state-of-the-art performance on ^1^H. The best performance provided by our model is at MAE values of 0.944 and 0.185 ppm for ^13^C and ^1^H, respectively. The baseline model without CST descriptors achieves slightly better performance than previous 2D graph neural networks. Incorporating knowledge from CST descriptors further reduces the MAE by around 12% on ^13^C and 5% on ^1^H. Both versions of the CST descriptor concatenation model show a similar level of prediction error. The two ensemble models provide the best prediction performance due to the elimination of potential bias from one single model.

**Table 2:** Performance of CSTShift Models on the NMRShiftDB2-DFT Data Set Compared with Other Reported Models^a^

**model**	**description**	^**13**^**C test MAE (ppm)**	^**1**^**H test MAE (ppm)**
HOSE^48^	nearest-neighbor search by atom environment encoding	2.850	0.330
GCN^48^	2D GNN	1.430	0.280
FCG^45^	2D GNN	1.355 ± 0.022	0.224 ± 0.002
scalable GNN^50^	2D GNN	1.271 ± 0.008	0.216 ± 0.001
			
CSTShift-noCST	3D GNN without concatenating CST descriptors	1.164 ± 0.005	0.206 ± 0.002
CSTShift-emb	3D GNN concatenating CST with input atomic embedding (Figure 2b)	1.019 ± 0.005	0.195 ± 0.001
CSTShift-out	3D GNN concatenating CST with message passing-updated embedding (Figure 2a)	1.043 ± 0.013	0.199 ± 0.001
CSTShift-emb (ensemble)	ensemble prediction of 5 CSTShift-emb models	**0.944**	**0.185**
CSTShift-out (ensemble)	ensemble prediction of 5 CSTShift-out models	0.959	0.189

a The best performance is in bold, and the second best one is underlined. Results of other baseline models are directly cited from corresponding works.

### Direct Test on the CHESHIRE Data Set

To further evaluate the prediction accuracy under extrapolation scenarios, we applied our CSTShift models to the CHESHIRE data set for external validation. The CHESHIRE data set was originally utilized to determine scaling factors for ^13^C and ^1^H by linearly fitting the DFT/GIAO-calculated isotropic shielding constants against the experimental chemical shifts. In this data set, 80 molecules in the test set were used for fitting, and 25 molecules in the probe set were used for evaluation.^17^ We compared three CSTShift variants with both DFT-LR (linear regression) and another 3D-based GNN model, ExpNN-ff^43^ on the probe set, which contains 107 ^13^C and 156 ^1^H nuclei. To build the data set with 3D structures and calculated shielding tensors, we optimized each molecule and then conducted single-point NMR calculations in chloroform at the level of B3LYP/6-31G(d). Following DFT-LR, we averaged the model output for nuclei with the same chemical environments as the final prediction, for example, three hydrogen atoms from a methyl group.

**Figure 3:**
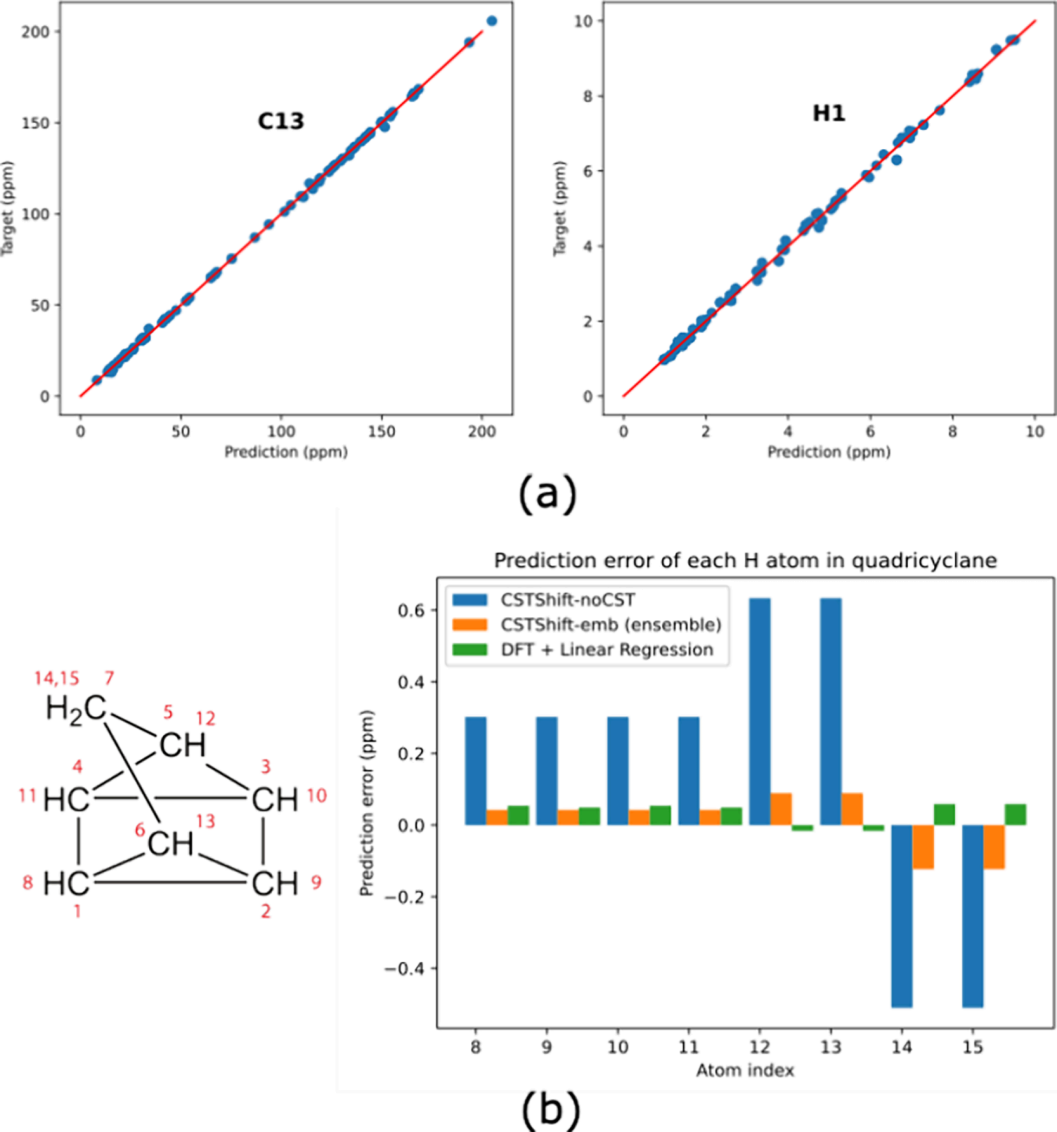
(a) Scatter plots between the predicted and experimental chemical shifts for ^13^C and ^1^H in the CHESHIRE data set by the CSTShift-emb ensemble model. (b) Structure and atom numbering of quadricyclane, with the prediction error comparison on its hydrogen atoms.

The performance on the CHESHIRE data set is summarized in Table 3, and the comparison between prediction values of the CSTShift-emb ensemble model and experimental chemical shifts is shown in Figure 3a. The two ensemble models with CST descriptors outperform both the linear model and ExpNN-ff on the ^13^C and ^1^H prediction. The prediction error of the CSTShift-noCST model is similar or larger than that of the DFT-LR method, while the precision difference between this and the model with CST descriptors is also larger than the prediction on NMRShiftDB2-DFT. As molecules in CHESHIRE data set are not covered in the NMRShiftDB2 training data set, the worse performance of CSTShift-noCST could be attributed to the lack of similar molecules during training. For example, we compared the prediction performance of the ^1^H chemical shifts of the quadricyclane in Figure 3b. This molecule has a bicyclic structure, which is not abundant in the ^1^H chemical shift training data set. As a result, the CSTShift-noCST model has worse predictions than the DFT methods for the ^1^H chemical shifts of the quadricyclane. However, the CST descriptors provide the correction for novel molecules, resulting in a large reduction in prediction error for the concatenation models. The combination with DFT-calculated NMR information enables more accurate prediction in situations where there is a lack of similar molecules in the training data set.

**Table 3:** Performance Comparison on the CHESHIRE Data Set^a^

**nuclei**	**metrics (ppm)**	**DFT-LR**(17)	**ExpNN-ff**(43)**(3D GNN)**	**CSTShift-noCST**	**CSTShift-emb (ensemble)**	**CSTShift-out (ensemble)**
^13^C	RMSE	2.769		2.679 ± 0.173	0.826	**0.818**
	MAE		1.500	1.276 ± 0.069	**0.504**	0.505
^1^H	RMSE	0.133		0.236 ± 0.052	**0.100**	0.101
	MAE			0.152 ± 0.018	0.079	**0.078**

a ExpNN-ff is a 3D GNN model, while the DFT-LR method uses the linear scaling for DFT calculation.

### Applications on the Structure Elucidation

**Figure 4:**
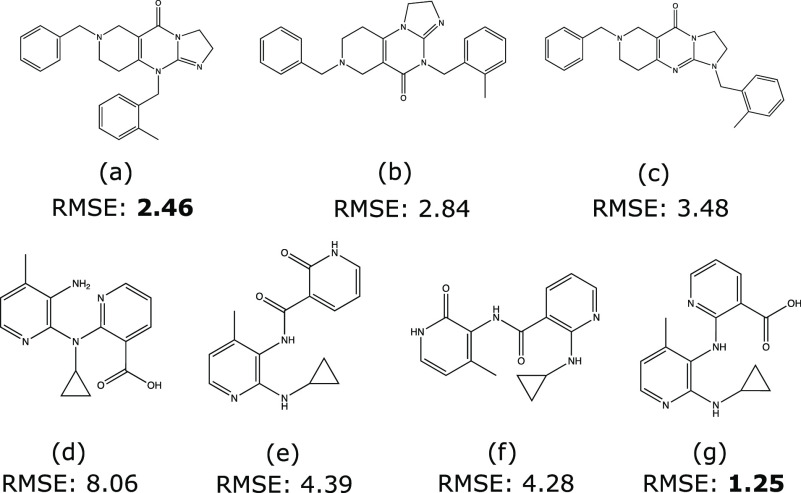
Isomer structures and CSTShift-emb ensemble prediction RMSE (unit: ppm) of TIC-10 (a–c) and NHP (d–g). (a) and (g) are the active isomers for TIC-10 and NHP, respectively.

Next, we applied our CSTShift model to elucidate the structures of two groups of organic compounds by predicting the ^13^C chemical shifts: TIC-10^73^ and nevirapine hydrolysis product (NHP).^74^ They represent important pharmaceutical compounds containing extended conjugated ring structures. Tumor necrosis factor (TNF)-related apoptosis-inducing ligand (TRAIL) is a cytokine that kills cancer cells but shows little toxicity against normal cells. The active pharmaceutical ingredient TIC-10 was reported to have the capability of inducing TRAIL expression. However, the structure was misassigned as (b) instead of the correct (a) shown in Figure 4.^76^ The structure misassignment could significantly slow the drug development process, as other isomers might lose pharmacological efficacy. Among three isomers shown in Figure 4, only (a) is capable of inducing TRIAL expression. The second test case, NHP, is the hydrolysis product of nevirapine, which is a powerful inhibitor of HIV-1 reverse transcriptase and contributes to the treatment of HIV-1 infection. Four structures (d)–(g) shown in Figure 4 were proposed from the mass spectroscopy and NMR results, where the computational structure elucidation is also required to identify the correct isomer.

Since both ensemble models show similar prediction accuracy across two experimental data sets, we only show the ^13^C chemical shift prediction RMSE of the CSTShift-emb ensemble model for both TIC-10 and NHP isomers in Figure 4. We prepared the 3D structure optimization and GIAO calculation for each isomer with the same process in DMSO at the level of B3LYP/6-31G(d). Compound (a) is selected as the predicted structure with the lowest RMSE among three TIC-10 isomers, which was reported as the pharmaceutically active isomer.^73^ For NHP isomers, our model also achieves success elucidation by selecting (g) with the lowest RMSE. Compared with the other two works conducting the same prediction task, our model has better prediction accuracy for the correct structures (a) and (g). Detailed comparisons are shown in Table S4 in the Supporting Information.

## Conclusions

In this work, we developed CSTShift, a 3D geometry-based GNN model that integrates atomic descriptors derived from DFT-calculated shielding tensors (CSTs) for accurate prediction of ^13^C and ^1^H chemical shifts in small molecules. Leveraging the extensive NMRShiftDB2 data set, which includes experimental chemical shifts for over 40,000 molecules, we conducted DFT optimization and GIAO calculations at the B3LYP/6-31G(d) level to assemble a comprehensive data set NMRShiftDB2-DFT that includes experimentally determined chemical shifts as well as DFT-optimized geometries and calculated shielding tensor (CST) descriptors. Our developed CSTShift model has yielded the best prediction accuracy across two experimental benchmarks, including the external CHESHIRE data set, achieving an MAE of 0.504 ppm for ^13^C and 0.078 ppm for ^1^H. Our model also successfully predicted the bioactive isomer in terms of lowest RMSE values among two groups of constitutional isomers, showcasing its capability in structure elucidation. As only one 3D structure of each molecule is used as input for our current model, providing multiple conformers to implement the Boltzmann-weighted average prediction has the potential to further improve the model’s robustness and applicability. Further improvement of the CSTShift model could also focus on bypassing or reducing the computational cost of DFT calculations for CST descriptors, which will be especially beneficial when scaling up to larger data sets or complex molecular systems.

## Data Availability

The 3D geometries for each compound in NMRShiftDB2-DFT, CHESHIRE, TIC-10, and NHP can be accessible at https://yzhang.hpc.nyu.edu/IMA, where the data processing, model building, and training procedure are also provided. RDKit 2022.09 version, PyTorch 1.12.1 version, and PyTorch Geometrics are used for model building/training, respectively.

## Supporting Information Available

Model hyperparameters and training setting (Table S1); detailed architecture of the CSTShift model (Figure S1); heavy atom number distributions of molecules in NMRShiftDB2-DFT (Figure S2); predicted ^13^C chemical shifts of the CHESHIRE data set (Table S2); predicted ^1^H chemical shifts of the CHESHIRE data set (Table S3); RMSE comparison for each isomer of TIC-10 and NHP groups (Table S4); atom numbering of TIC-10 isomers (Figure S3); difference between CSTShift-emb ensemble model prediction and experimental ^13^C chemical shifts of each isomer in TIC-10 (Figure S4); atom numbering of NHP isomers (Figure S5); difference between CSTShift-emb ensemble model prediction and experimental ^13^C chemical shifts of each isomer in NHP (Figure S6) (PDF)
