# Supplementary material for: Accurate Prediction of NMR Chemical Shifts: Integrating DFT Calculations with Three-Dimensional Graph Neural Networks
Source: J Chem Theory Comput. 2024 Jun 6;20(12):5250–8. doi: 10.1021/acs.jctc.4c00422 (PMC11209944; doi:10.1021/acs.jctc.4c00422)
Supplement: Supplementary file 1 — ct4c00422_si_001.pdf [file ct4c00422_si_001.pdf]

Supporting Information

Accurate Prediction of NMR Chemical Shifts: Integrating DFT calculations with 3D Graph Neural Networks

Chao Han[1†], *Dongdong Zhang*[1†], *Song Xia*[1] *and Yingkai Zhang*[1,2,3*]

[1]Department of Chemistry, New York University, New York, New York 10003, United States

[2]Simons Center for Computational Physical Chemistry at New York University, New York, New York 10003, United States

[3]NYU-ECNU Center for Computational Chemistry at NYU Shanghai, Shanghai 200062, China

[†]C.H. and D.Z. contributed equally.

Table S1. Model hyperparameters and training setting.

Hyper parameters	Values
# modules	5
# Embedding feature	512
Batch size	32
# Max epochs	1000
Initial learning rate	0.001
Learning rate scheduler	ReduceLROnPlateau(factor=0.5, patience=30, eps=5e-8)
Optimizer	AMSGrad(betas=(0.9, 0.99), weight decay=0)
Molecular graph edge cutoff	10Å

Figure S1. Detailed architecture of CSTShift model. (A) The general structure of the CSTShift model. CONCAT in the dash box shows different implementations of concatenation with CST descriptors. In CSTShift-emb, CONCAT is used after the embedding layer. In CSTShift-out, the CONCAT is used after the message passing layers and before the output layer. No CONCAT will be used in CSTShift-noCST as a baseline model. (B) Message Passing (MP) module. Square symbols represent the input for the layers, ∘ represents Hadamard products. (C) Output module. (D) Interaction module. (E) Residual module.

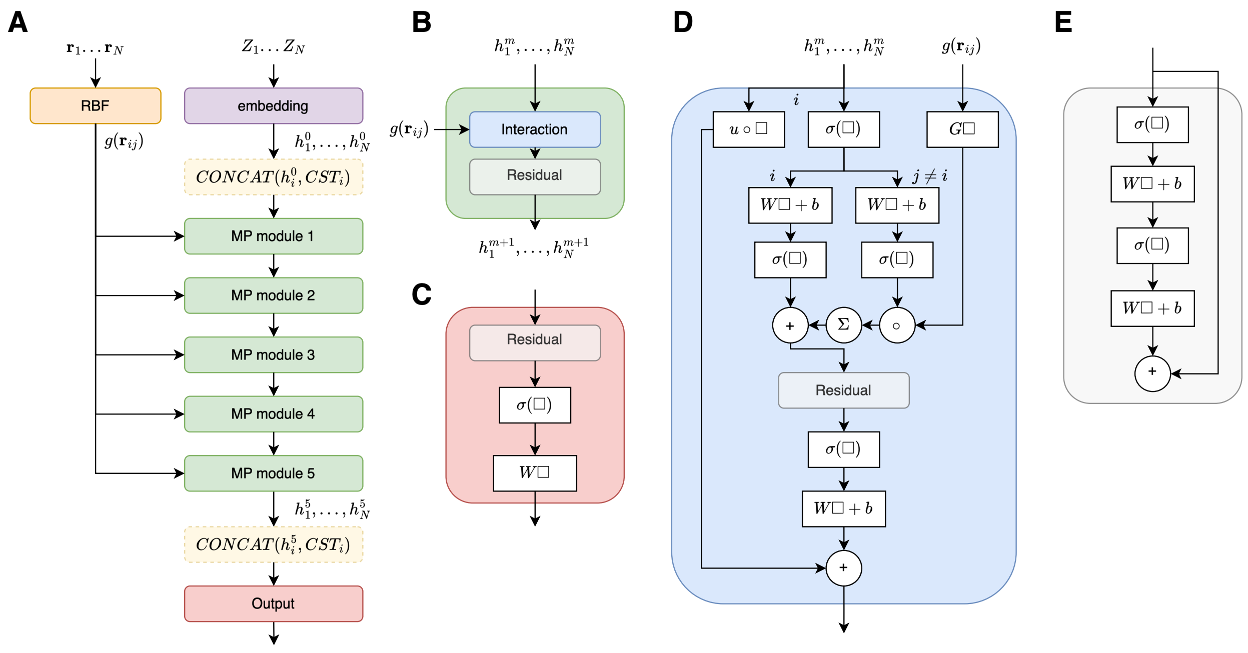

Figure S2. The total atom number and heavy atom number distributions of molecules in NMRShiftDB2-DFT training datasets.

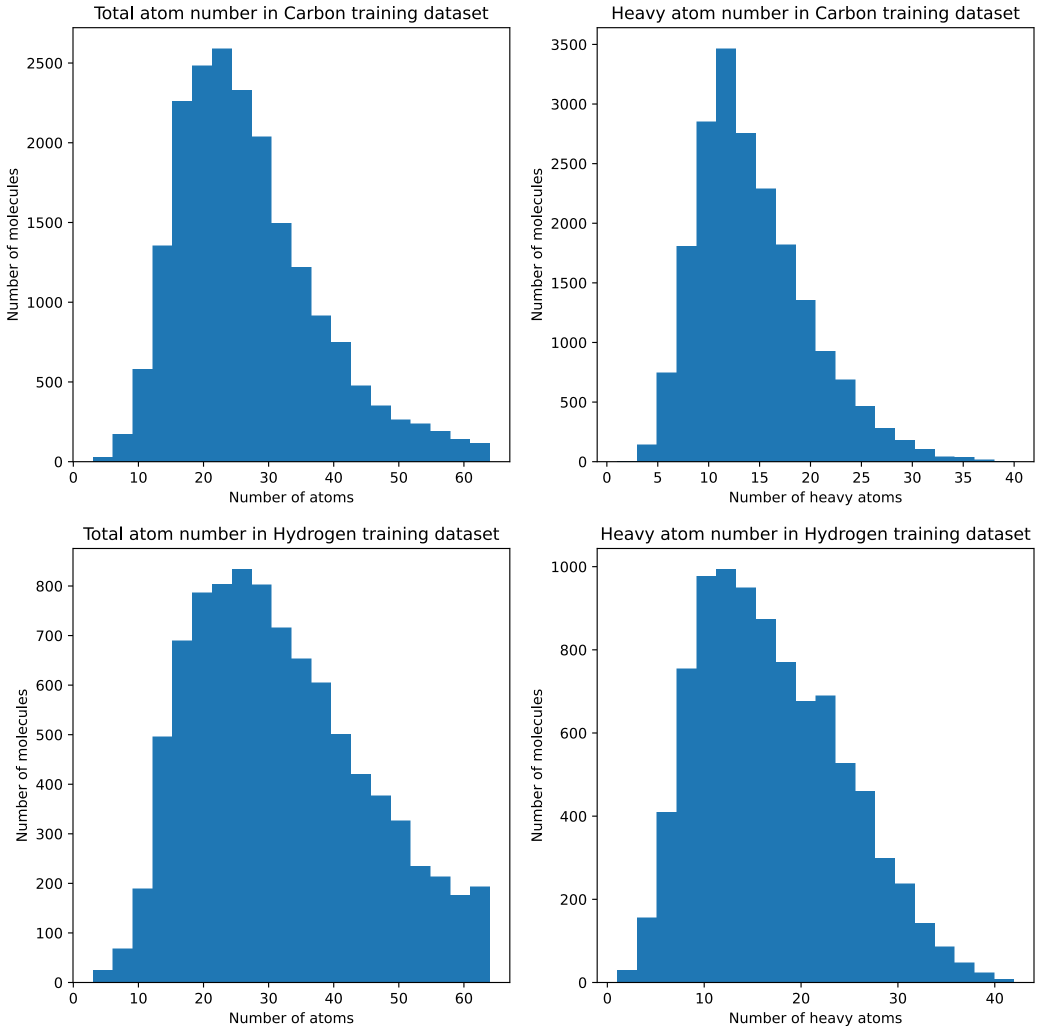

Table S2. The predicted ^{13}C chemical shifts of CHESHIRE dataset. Molecule ID, atom index and experimental shift are from the probe set database on CHESHIRE dataset website (http://cheshirenmr.info/index.htm). The prediction is provided by our CSTShift-emb ensemble model.

Molecule ID	Atom index	Prediction	Experimental shift
0	0	29.614	30.48
0	2	129.378	128.9
1	1	193.633	194.04
1	2	134.59	134.61
1	3	154.069	154.32
1	4	18.396	18.61
2	0	13.456	13.21
2	1	115.872	115.93
2	2	141.836	141.67
2	3	33.982	36.99
2	4	22.017	21.52
2	5	22.017	21.52
2	6	15.363	13.06
3	1	165.163	164.58
3	2	165.163	164.58
3	3	136.615	136.76
3	4	136.615	136.76
4	1	75.267	75.42
4	2	75.267	75.42
4	3	126.316	126.34
4	4	126.316	126.34
5	1	149.767	149.94
5	2	149.767	149.94
5	3	123.605	123.55
5	4	123.605	123.55
5	5	135.961	135.89
6	1	154.228	154.11
6	2	144.414	144.85
6	4	141.869	141.9
6	5	144.19	143.9
6	6	21.399	21.53
7	0	104.736	104.79
7	1	150.028	150.62
7	2	32.352	32.08

7	3	32.352	32.08
7	4	16.317	16.76
8	0	93.835	94.28
8	3	66.917	66.94
8	4	66.917	66.94
8	5	26.479	26.64
9	0	101.6	101.24
9	1	18.707	18.8
9	4	52.673	52.31
9	5	52.673	52.31
10	1	139.207	139.5
10	2	125.29	125.14
10	3	126.772	126.86
10	4	123.648	123.03
10	5	15.151	14.95
11	0	8.099	8.77
11	1	111.003	109.35
11	2	111.003	109.35
12	0	155.59	155.92
12	2	64.901	65.05
12	3	64.901	65.05
13	1	26.086	25.5
13	2	26.086	25.5
13	3	14.457	14.8
13	4	14.457	14.8
14	0	119.243	119.33
14	1	119.243	119.33
14	2	115.453	114.26
14	3	115.453	114.26
15	0	168.381	168.39
15	1	44.556	44.36
15	2	67.873	68.12
15	4	20.16	20.58
16	1	40.769	40.53
16	2	40.769	40.53
16	3	119.409	119.36
17	0	118.833	117.49
17	1	130.59	130.4
17	2	47.572	47.12

17	4	133.743	132.02
18	1	166.001	166.14
18	3	166.001	166.14
18	5	166.001	166.14
19	0	166.172	165.18
19	2	151.539	147.66
19	3	41.934	42.4
19	5	86.806	87.06
20	0	42.792	42.5
20	1	23.569	23.44
20	2	23.569	23.44
20	3	23.569	23.44
20	4	204.958	205.83
21	0	115.656	113.64
21	1	142.143	142.46
21	2	139.942	139.82
21	3	114.028	116.75
21	4	18.509	17.85
22	0	54.309	54.14
22	1	30.842	30.8
22	2	30.842	30.8
22	3	30.842	30.8
22	5	154.241	153.39
23	1	109.584	109.62
23	2	144.194	144.44
23	4	65.229	65.4
23	5	13.658	13.73
24	0	15.909	14.71
24	1	15.909	14.71
24	2	15.909	14.71
24	3	15.909	14.71
24	4	22.189	22.99
24	5	22.189	22.99
24	6	30.775	31.98

Table S3. The Predicted ^{1}H chemical shifts of CHESHIRE dataset. Molecule ID, atom index and experimental shift are from the probe set database on CHESHIRE dataset website (http://cheshirenmr.info/index.htm). The prediction is provided by our CSTShift-emb ensemble model.

Molecule ID	Atom index	Prediction	Experimental shift
0	4	3.246	3.325
0	5	3.246	3.325
0	6	3.246	3.325
1	5	9.508	9.497
1	6	6.143	6.145
1	7	6.96	6.88
1	8	1.991	2.032
1	9	1.991	2.032
1	10	1.991	2.032
2	7	1.616	1.56
2	8	1.616	1.56
2	9	1.616	1.56
2	10	5.191	5.222
2	11	2.143	2.221
2	12	0.984	0.978
2	13	0.984	0.978
2	14	0.984	0.978
2	15	0.984	0.978
2	16	0.984	0.978
2	17	0.984	0.978
2	18	1.616	1.56
2	19	1.616	1.56
2	20	1.616	1.56
3	7	7.024	7.048
3	8	7.024	7.048
4	5	4.518	4.63
4	6	4.518	4.63
4	7	4.518	4.63
4	8	4.518	4.63
4	9	5.898	5.89
4	10	5.898	5.89
5	6	8.607	8.593
5	7	8.607	8.593

5	8	7.287	7.231
5	9	7.287	7.231
5	10	7.685	7.617
6	7	2.566	2.566
6	8	2.566	2.566
6	9	2.566	2.566
6	10	8.476	8.568
6	11	8.406	8.376
6	12	8.563	8.453
7	5	4.821	4.685
7	6	4.821	4.685
7	7	2.591	2.685
7	8	2.591	2.685
7	9	2.591	2.685
7	10	2.591	2.685
7	11	1.925	1.928
7	12	1.925	1.928
8	6	4.684	4.853
8	7	4.684	4.853
8	8	3.859	3.912
8	9	3.859	3.912
8	10	3.859	3.912
8	11	3.859	3.912
8	12	1.691	1.78
8	13	1.691	1.78
9	6	4.424	4.568
9	7	1.25	1.281
9	8	1.25	1.281
9	9	1.25	1.281
9	10	3.345	3.309
9	11	3.345	3.309
9	12	3.345	3.309
9	13	3.345	3.309
9	14	3.345	3.309
9	15	3.345	3.309
10	6	6.668	6.754
10	7	6.759	6.89
10	8	6.94	7.07
10	9	2.35	2.495

10	10	2.35	2.495
10	11	2.35	2.495
11	5	3.771	3.604
11	6	3.771	3.604
12	6	4.427	4.541
12	7	4.427	4.541
12	8	4.427	4.541
12	9	4.427	4.541
13	5	2.612	2.547
13	6	2.612	2.547
13	7	2.612	2.547
13	8	2.612	2.547
13	9	1.258	1.256
13	10	1.258	1.256
13	11	1.258	1.256
13	12	1.258	1.256
13	13	1.258	1.256
13	14	1.258	1.256
14	6	6.637	6.295
14	7	6.637	6.295
15	6	3.25	3.083
15	7	3.364	3.56
15	8	4.819	4.704
15	9	1.435	1.563
15	10	1.435	1.563
15	11	1.435	1.563
16	5	2.724	2.853
16	6	2.724	2.853
16	7	2.724	2.853
16	8	2.724	2.853
16	9	2.724	2.853
16	10	2.724	2.853
17	6	5.296	5.41
17	7	5.303	5.3
17	8	5.966	5.83
17	9	3.938	4.147
17	10	3.938	4.147
18	6	9.064	9.231
18	7	9.064	9.231

18	8	9.064	9.231
19	6	3.907	3.897
19	7	3.907	3.897
19	8	4.732	4.877
19	9	4.748	4.494
20	6	1.143	1.08
20	7	1.143	1.08
20	8	1.143	1.08
20	9	1.143	1.08
20	10	1.143	1.08
20	11	1.143	1.08
20	12	1.143	1.08
20	13	1.143	1.08
20	14	1.143	1.08
20	15	9.409	9.48
21	5	5.041	4.99
21	6	5.041	4.99
21	7	6.319	6.44
21	8	5.141	5.19
21	9	5.103	5.06
21	10	1.888	1.85
21	11	1.888	1.85
21	12	1.888	1.85
22	6	1.329	1.446
22	7	1.329	1.446
22	8	1.329	1.446
22	9	1.329	1.446
22	10	1.329	1.446
22	11	1.329	1.446
22	12	1.329	1.446
22	13	1.329	1.446
22	14	1.329	1.446
23	7	4.37	4.412
23	8	4.37	4.412
23	9	1.364	1.394
23	10	1.364	1.394
23	11	1.364	1.394
24	7	1.52	1.478
24	8	1.52	1.478

24	9	1.52	1.478
24	10	1.52	1.478
24	11	1.434	1.345
24	12	1.434	1.345
24	13	1.89	2.013
24	14	1.89	2.013

Table S4. RMSE comparison for each isomer of TIC-10 and NHP groups. a is the correct isomer structure of TIC-10 and d is the correct isomer structure of NHP. The first baseline model prediction is from DFT-GIAO calculation with linear scaling[1]. The second baseline model is a machine learning method based on Deep Neural Network (DNN) and molecular descriptors[2].

	**TIC-10**			**NHP**			
isomer	a	b	c	a	b	c	d
DFT-GIAO/scaling	2.80	3.14	5.57	10.78	6.16	5.70	2.37
DNN + Molecular Descriptors	0.83	2.16	4.26	10.46	6.01	5.54	2.37
CSTShift-out ensemble	**2.36**	2.67	3.48	8.28	4.65	4.32	**1.50**
CSTShift-emb ensemble	**2.46**	2.84	3.48	8.06	4.39	4.28	**1.25**

Figure S3. The atom numbering of TIC-10 isomers.

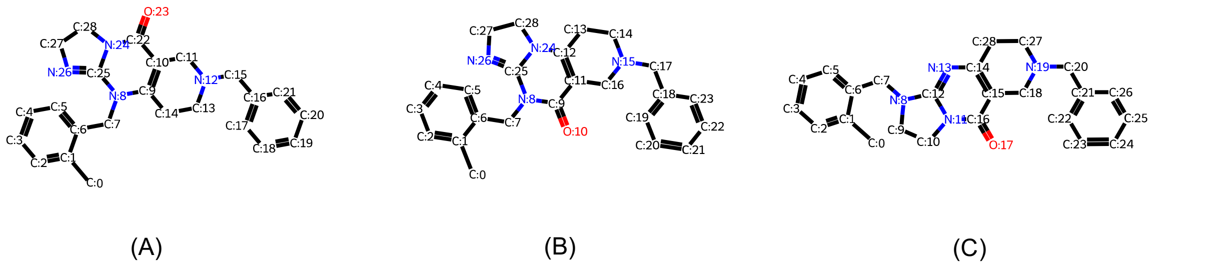

Figure S4. The difference between CSTShift ensemble model predictions and experimental ^{13}C chemical shifts of each isomer in TIC-10.

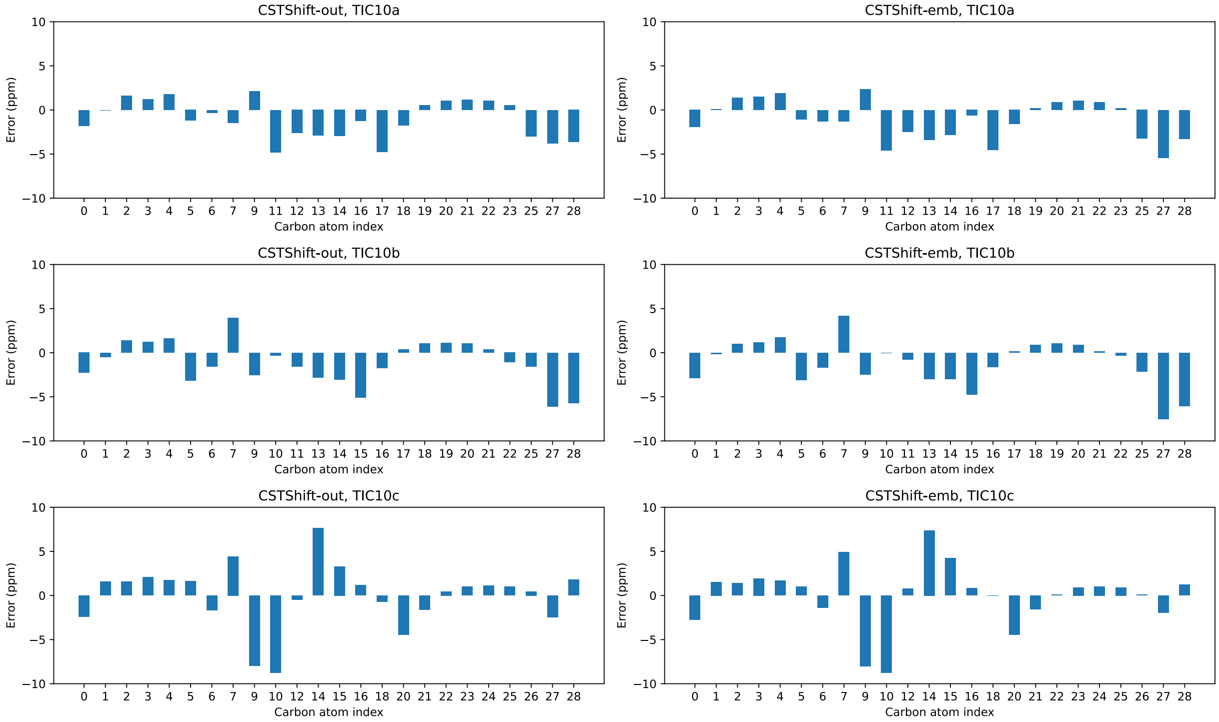

Figure S5. The atom numbering of NHP isomers.

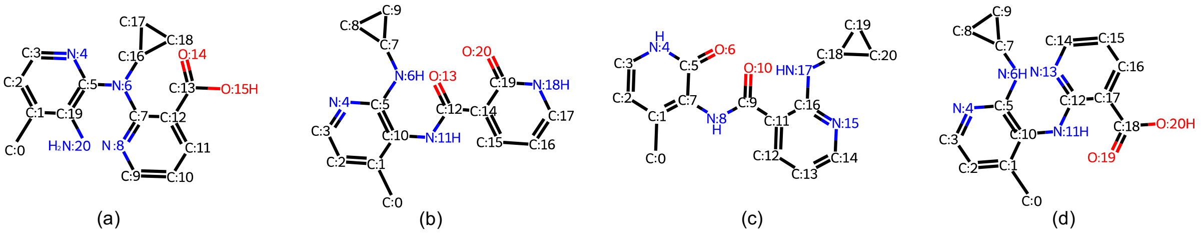

Figure S6. The difference between CSTShift ensemble model predictions and experimental ^{13}C chemical shifts of each isomer in NHP.

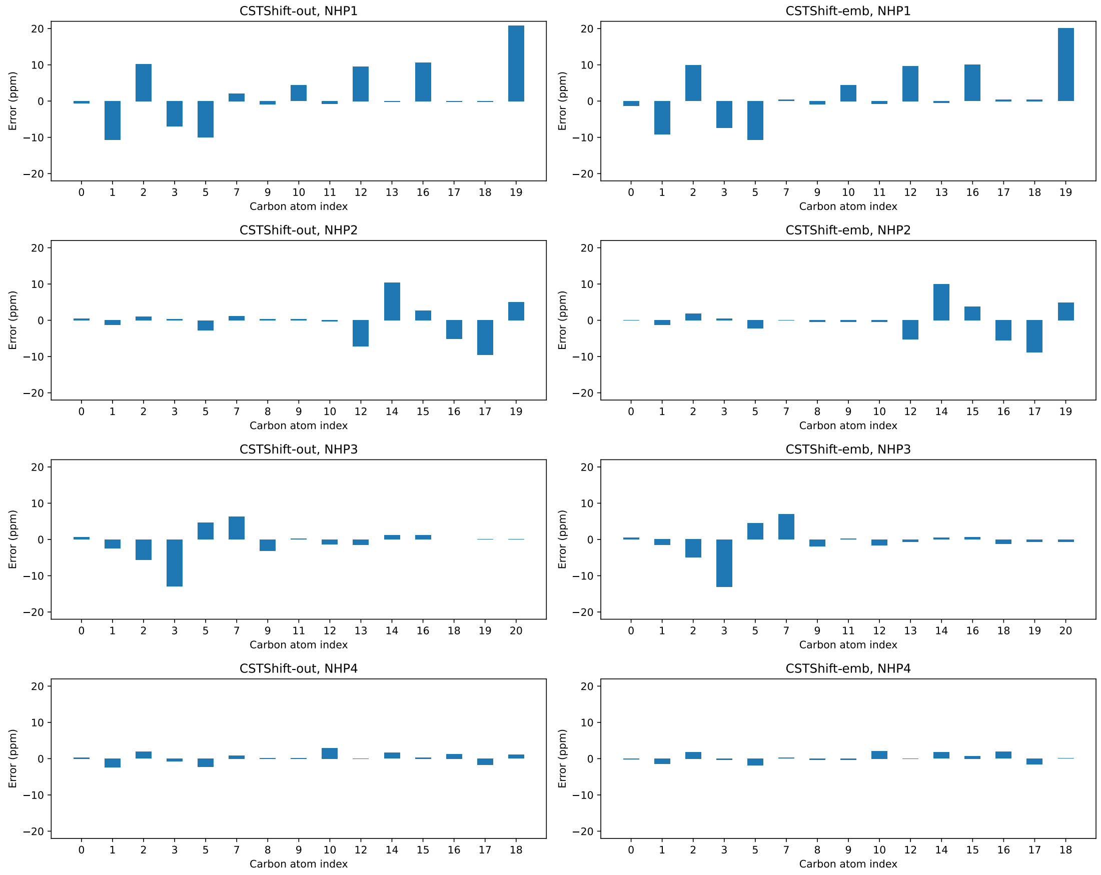

References

(1) Xin, D.; Sader, C. A.; Chaudhary, O.; Jones, P.-J.; Wagner, K.; Tautermann, C. S.; Yang, Z.; Busacca, C. A.; Saraceno, R. A.; Fandrick, K. R.; Gonnella, N. C.; Horspool, K.; Hansen, G.; Senanayake, C. H. Development of a 13C NMR Chemical Shift Prediction Procedure Using B3LYP/Cc-pVDZ and Empirically Derived Systematic Error Correction Terms: A Computational Small Molecule Structure Elucidation Method. *J. Org. Chem.* **2017**, *82* (10), 5135–5145. https://doi.org/10.1021/acs.joc.7b00321.

(2) Gao, P.; Zhang, J.; Peng, Q.; Zhang, J.; Glezakou, V.-A. General Protocol for the Accurate Prediction of Molecular 13C/1H NMR Chemical Shifts via Machine Learning Augmented DFT. *J. Chem. Inf. Model.* **2020**, *60* (8), 3746–3754. https://doi.org/10.1021/acs.jcim.0c00388.
